# 

**Published:** 1887-12

**Authors:** 


					﻿/olume LV.]
DECEMBER, 1887.
[Number 6

/'ABDK^lL i
1 Journal
i EXAMINER
EDITOR:
S. J. JONES, M.D., LL.D
I ) f I IV !■•■■> I l u 1 l 11 • • ■	r A	'• //
(jLARK & EONGLLI \U)Qilv\.QU9]
/....... publishers.............................  i
Entered at the Poet Office at Chicago, t,or transmission through the mailt at tacand-class matter.
				

